# Association between neutrophil-to-lymphocyte ratio and left ventricular diastolic dysfunction in patients with type 2 diabetes mellitus

**DOI:** 10.3389/fendo.2024.1499713

**Published:** 2025-01-10

**Authors:** Xueyang Yang, Yinze Shi, Huan Zhang, Liying Huang, Jiaoyue Zhang, Jie Min, Lulu Chen

**Affiliations:** ^1^ Department of Endocrinology, Union Hospital, Tongji Medical College, Huazhong University of Science and Technology, Wuhan, China; ^2^ Hubei Provincial Clinical Research Center for Diabetes and Metabolic Disorders, Wuhan, China

**Keywords:** neutrophil-to-lymphocyte ratio, type 2 diabetes mellitus, left ventricular diastolic dysfunction, heart failure, inflammatory biomarkers

## Abstract

**Background:**

Diabetes has become a global pandemic, posing a sustained threat to human health, primarily due to its associated complications. Left ventricular diastolic dysfunction (LVDD) is a prevalent cardiac complication among patients with diabetes. Since most patients are asymptomatic and lack relevant biomarkers, LVDD has not attracted significant attention from clinicians. The neutrophil-to-lymphocyte ratio (NLR) is a widely studied inflammation biomarker that has been suggested to be linked to various medical conditions, including cardiac diseases. However, its association with LVDD among patients with type 2 diabetes mellitus (T2DM) has not been explored.

**Aim:**

To clarify the relationship between NLR and LVDD among patients with type 2 diabetes.

**Methods:**

We conducted a cross-sectional study using medical records from 855 patients diagnosed with T2DM who were admitted to the Endocrinology department at Wuhan Union Hospital. According to the ASE/EACVI 2016 recommendations, these patients were categorized into two groups based on sonographic parameters: patients with normal left ventricular diastolic function (the non-LVDD group) and patients with LVDD (the LVDD group). NLR values were calculated and divided into three different levels. Statistical analysis was conducted to evaluate the correlation between NLR levels and the prevalence of LVDD.

**Results:**

The prevalence of LVDD among hospitalized patients with T2DM in our study was 47.8% (409/855). The mean NLR value of the LVDD group was significantly higher compared with the non-LVDD group [1.60 (1.24-2.05) vs 1.85 (1.44-2.31), P<0.001]. The prevalence of LVDD in the three different NLR levels was 35.51% (76/214), 49.27% (203/412), and 56.77% (130/229), respectively. Unjustified logistic analysis showed that NLR levels were positively associated with the prevalence of LVDD (P <0.001). Compared to the low level of NLR, the unadjusted odds ratios (OR) of LVDD at the medium and high levels were 1.764 (1.255-2.478, P=0.001) and 2.384 (1.626-3.497, P<0.001), respectively (P for trend <0.001).

**Conclusion:**

Our findings suggest that the NLR is a potential indicator for assisting clinicians in identifying LVDD in patients with T2DM. Patients with elevated NLR levels may be at a greater risk of developing LVDD than those with lower NLR levels, which may require attention and interventions to prevent patients from progressing into heart failure.

## 1 Introduction

According to the latest reports by the World Health Organization (WHO), the global prevalence of diabetes in adults increased from 7% in 1990 to 14% in 2022, with patients exceeding 800 million—representing more than a fourfold increase (1). Heart failure (HF) is a frequent complication of diabetes, affecting up to 22% of individuals with the condition in the American population (2). However, left ventricular diastolic dysfunction (LVDD), diagnosed by echocardiography, is considered a myocardial functional impairment phenotype of diabetic cardiomyopathy and has a prevalence of up to 43% in diabetic patients (3). LVDD is recognized as an early stage of heart failure, also referred to as Stage B heart failure, where patients exhibit structural or functional cardiac abnormalities without overt clinical symptoms or signs (4). However, there is a lack of serum biomarkers for identifying LVDD in high-risk individuals, as conventional heart failure-related biomarkers, such as serum brain natriuretic peptides (BNP), typically remain within the normal range during the early stages of HF. Therefore, it is imperative to explore novel biomarkers capable of identifying LVDD to prevent the onset and progression of overt HF.

Inflammation serves both as a cause and a consequence of HF, playing a pivotal role in its pathogenesis and progression (5, 6). Comorbidities frequently associated with HF, such as diabetes, obesity, and chronic kidney disease, contribute to a state of chronic low-grade inflammation. The neutrophil-to-lymphocyte ratio (NLR), derived from a complete blood count, is a marker of systemic inflammation. It has been confirmed to be linked with multiple inflammatory conditions, such as cardiovascular diseases, malignancies, infections, and hemorrhagic disorders (7). Previous clinical studies have primarily examined the predictive value of NLR for adverse disease outcomes, concluding that elevated NLR levels may signify a more severe disease prognosis in patients (8–11). The relationship between NLR and LVDD in patients with T2DM remains unexplored, with no published studies addressing this issue. Examining these relationships may assist clinicians in identifying patients at elevated risk of LVDD.

## 2 Methods

### 2.1 Study design and participants

Clinical data were collected from 855 patients diagnosed with T2DM who were hospitalized at the Endocrinology Department of Wuhan Union Hospital between January 2019 and January 2021. Detailed clinical information included the patient’s age, gender, BMI, blood pressure, smoking history, family history of diabetes, duration of diabetes, HbA1c, fast blood glucose, liver function, kidney function, lipid profile, uric acid, neutrophil and lymphocyte count. All patients included in the study were of Han ethnicity. The inclusion criteria were as follows: age>18 years old; a diagnosis of type 2 diabetes; access to complete blood count and echocardiography examination; ejection fraction (EF) greater than 50%. T2DM was defined according to the 2023 ADA Standards of Care in Diabetes (12). The following criteria were used for exclusion: patients with previous histories of coronary heart disease, valvular heart diseases, or known clinical heart failure; patients with severe liver or kidney dysfunction; patients with acute infectious diseases; patients diagnosed with hematological diseases; and patients with a history of malignant tumors. Heart failure (HF) was diagnosed according to the 2022 AHA/ACC/HFSA Guidelines (13). Severe hepatic or renal dysfunction was characterized by ALT levels surpassing three times the upper limit of normal or an eGFR below 30 mL/min/1.73 m². Acute infectious and hemorrhagic diseases were identified through pertinent clinical manifestations and hematological indicators.

### 2.2 Evaluation of left ventricular function

Echocardiographic tests were conducted using echocardiographic systems (GE Vivid 7; Vingmed; Philips IE33 and Philips EPIQ 7C) with 3-8 MHz transducers. Two experienced ultrasonography specialists identified signs of diastolic dysfunction based on the E/A ratio values of the mitral and septal basal regions. The left ventricular end-diastolic diameter (LVEDD), left atrial diameter (LAD), interventricular septum thickness (IVST), and left ventricular ejection fraction (LVEF) were measured. LVEF was determined using the biplane Simpson’s approach. Peak velocities in the early (E-wave) (MVE) and late (A-wave) (MVA) phases of the mitral inflow pattern were determined using apical four-chamber images. According to the ASE/EACVI 2016 recommendations, patients can be identified as LVDD if the following criteria are met: average E/e ratio > 14 or E/e’ ratio < 14 with an E/A ratio < 0.8 (14).

### 2.3 NLR calculating and grouping

The NLR values were calculated using the formula NLR = N/L from the absolute peripheral granulocyte (N; 109/Liter) and lymphocyte (L; 109/Liter) blood counts. Our study defined the first 25% of NLR values as the low-level group, the middle 50% as the moderate-level group, and the last 25% as the high-level group.

### 2.4 Statistics analysis

The analysis was conducted using IBM SPSS Statistics, Version 22 (IBM Corporation, Armonk, NY, USA). A two-sided P value of <0.05 was considered significant. Continuous variables were expressed as mean ± SD or median (interquartile range, IQR), while categorical variables were presented as percentages (%) based on the data distribution and types. Logistic regression analysis assessed the trend of variable changes across different NLR levels, providing ORs and P values for adjusted models. The data were summarized as ORs and regression coefficients (95% CI).

## 3 Results

### 3.1 Baseline characteristics of the enrolled T2DM patients C

Our data analysis involved 855 hospitalized patients diagnosed with T2DM, as detailed in **Table 1**. The study population had a mean age of 53.39 ± 11.37 years, with females comprising 30.9% of the total patients. The average HbA1c level was 9.20 ± 3.62%, and the mean BMI was 25.28 ± 3.69 kg/m². Patients were divided into two groups based on the evaluation of left ventricular diastolic function: the normal group (n=466, non-LVDD group) and the diastolic dysfunction group (LVDD group, n=409). In comparison to the non-LVDD group, patients in the LVDD group were older (58.75 ± 8.10 years old vs. 48.64 ± 11.86 years old, P<0.001), had a longer duration of diabetes (8.00 (3.00-15.00) vs. 5.00 (0.94-10.00) years, P<0.001), lower estimated glomerular filtration rate (eGFR) (104.97 (96.31-116.00) vs. 95.43 (77.23-102.87) ml/min/1.73m2, P<0.001), and higher systolic blood pressure (SBP) (133.4 ± 17.66 vs. 129.20 ± 16.71 mmHg, P<0.001). No significant differences were observed in fasting blood glucose (FBG), diastolic blood pressure, and lipid profiles (all P values >0.05). A complete blood cell count was obtained from all patients, and the NLR values were calculated. The results showed significant differences in NLR values between the two groups. The NLR of LVDD group patients was significantly higher compared to the non-LVDD group (1.85 (1.44-2.31) vs 1.60 (1.24-2.05), P<0.001).

**Table 1:** Basic characteristics of patients with type 2 diabetes grouped by left ventricular function.

	Overall (n=855)	non-LVDD (n=446)	LVDD (n=409)	P
Sex, n (Female%)	264 (30.9)	132 (29.6)	132 (32.3)	0.397
Age (years)	53.39 ± 11.37	48.64 ± 11.86	58.75 ± 8.10	<0.001
Course of diabetes(years)	6.00 (1.00-13.00)	5.00 (0.94-10.00)	8.00 (3.00-15.00)	<0.001
Family diabetes history (%)	394 (46.1)	215 (48.2)	179 (43.8)	0.193
Hypertension history (%)	120 (14.0)	60 (13.5)	60 (14.7)	0.609
Smoking (%)	375 (43.9)	205 (46.0)	170 (41.6)	0.195
Height (mm)	167.26 ± 7.80	168.02 ± 7.76	166.43 ± 7.81	0.003
Weight (kg)	71.02 ± 13.32	72.21 ± 14.93	69.83 ± 11.14	0.009
BMI (kg/m2)	25.28 ± 3.69	25.45 ± 4.14	25.13 ± 3.09	0.205
SBP (mmHg)	131.15 ± 17.25	129.20 ± 16.71	133.42 ± 17.66	<0.001
DBP (mmHg)	81.36 ± 11.74	80.89 ± 11.22	82.01 ± 12.34	0.163
AST (U/L)	20.00 (17.00,26.00)	21.00 (17.00-27.00)	20.00 (17.00-26.00)	0.548
ALT (U/L)	22.00 (16.00,34.00)	23.00 (16.00-35.00)	21.00 (16.00-31.50)	<0.001
TBA (mmol/L)	3.35 (2.10,5.18)	3.15 (1.90-5.20)	3.65 (2.40-5.10)	0.045
SCr (umol/L)	67.10 (56.05,80.40)	65.10 (54.40-74.40)	71.00 (58.10-88.58)	<0.001
BUN (mmol/L)	5.69 (4.58,7.00)	5.38 (4.35-6.56)	6.10 (4.94-7.58)	<0.001
eGFR (ml/min)	100.79 (89.33, 109.86)	104.97 (96.31-116.00)	95.43 (77.23-102.87)	<0.001
UA (umol/L)	331.40 (271.50,401.00)	335.20 (268.50-399.10)	327.75 (277.60-405.25)	0.647
TC (mmol/L)	4.64 ± 1.15	4.69 ± 1.13	4.60 ± 1.18	0.257
TG (mmol/L)	1.48 (1.02,2.32)	1.51 (1.04-2.55)	1.43 (1.02-2.12)	0.179
HDL-C (mmol/L)	1.09 ± 0.37	1.08 ± 0.40	1.11 ± 0.33	0.188
LDL-C (mmol/L)	2.74 ± 0.95	2.77 ± 0.95	2.72 ± 0.96	0.395
FBG (mmol/L)	7.84 ± 2.43	7.86 ± 2.35	7.74 ± 2.27	0.479
HbA1c (%)	9.20 ± 3.62	9.50 ± 4.58	8.86 ± 2.16	0.011
Neutrophil count (10^9^/l)	3.31 (2.70,4.09)	3.29 (2.67-4.08)	3.42 (2.71-4.10)	0.247
Lymphocyte count (10^9^/l)	2.00 ± 0.63	2.10 ± 0.64	1.87 ± 0.59	<0.001
NLR	1.69 (1.31,2.20)	1.60 (1.24-2.05)	1.85 (1.44-2.31)	<0.001

Data were given as mean (SD) or median (IQR), or number (percent), as appropriate. SBP, systolic blood pressure; DBP, diastolic blood pressure; AST, aspartate aminotransferase; ALT, alanine aminotransferase; TBA, total bile acid; FBG, fasting blood glucose; HDL-cholesterol, high-density lipoprotein cholesterol; LDL-cholesterol, low-density lipoprotein cholesterol; HbA1c, hemoglobin A1c; BUN, blood urea nitrogen; SCr, serum creatinine; BMI, body mass index; UA, uric acid; NLR, neutrophil-to-lymphocyte ratio; LVDD, left ventricular diastolic dysfunction.

### 3.2 Logistic regression analysis of clinical parameters and LVDD prevalence

As shown in **Table 2**, univariate regression analysis showed that the LVDD prevalence was significantly associated with the patient’s age, course of diabetes, HbA1c, SBP, creatinine, eGFR, and NLR (all P values<0.05), while no significant relationships were found regarding FBG, BMI, DBP, uric acid, and TBA (all P values>0.05). Concerning the blood lipid profile, only triglycerides demonstrated a slight but statistically significant association with LVDD prevalence (P=0.047). By performing multivariate regression analysis, the following variables remained significantly associated with LVDD: patient’s age, SBP, and creatinine level, while NLR, course of diabetes, and HbA1c did not show an independent association with LVDD (all P values >0.05).

**Table 2:** Univariate and multivariate logistic regression analyses on the relationship between clinical parameters and prevalence of LVDD.

Characteristics	Univariate analysis			Multivariate analysis		
	OR	95%CI	P	OR	95%CI	P
Sex	0.882	0.66-1.179	0.397	1.126	0.783-1.619	0.521
Age	1.107	1.088-1.126	<0.001	1.113	1.091-1.136	<0.001
Course of diabetes	1.051	1.031-1.072	<0.001	0.983	0.96-1.007	0.155
Smoking	0.836	0.638-1.096	0.195	–	–	–
BMI	0.977	0.941-1.013	0.205	1.043	0.996-1.093	0.073
SBP	1.014	1.006-1.023	<0.001	1.015	1.005-1.025	0.002
DBP	1.008	0.997-1.020	0.163	–	–	–
NLR	1.326	1.138-1.546	<0.001	1.033	0.953-1.119	0.431
HbA1c	0.913	0.858-0.970	0.003	0.968	0.907-1.034	0.334
AST	0.989	0.980-0.998	0.020	–	–	–
ALT	0.987	0.981-0.993	<0.001	–	–	–
TBA	1.005	0.977-1.033	0.744	–	–	–
SCr	1.014	1.009-1.020	<0.001	1.008	1.003-1.014	0.005
BUN	1.009	0.987-1.031	0.428	–	–	–
eGFR	0.967	0.960-0.974	<0.001	–	–	–
UA	1.001	0.999-1.002	0.332	–	–	–
TC	0.934	0.831-1.051	0.257	–	–	–
TG	0.935	0.875-0.999	0.047	–	–	–
HDL-C	1.286	0.880-1.879	0.188	–	–	–
LDL-C	0.94	0.816-1.083	0.395	–	–	–
FBG	0.979	0.922-1.039	0.479	–	–	–

### 3.3 Baseline data grouped by NLR levels

NLR values were divided into three groups using the following methods: the lowest 25% were considered as the low-level group (0.601,1.316), the middle 50% as the medium-level group (1.316,2.156), and the highest 25% as the high-level group (2.156,15.622). The prevalence of LVDD was 35.51% (76/214) for the low-level NLR group, 49.27% (203/412) for the medium-level group, and 56.77% (130/229) for the high-level group (P < 0.001), respectively. Baseline data showed that the patient’s sex, age, course of diabetes, serum creatinine (SCr), and eGFR were statistically different across the three groups. Echocardiographic parameters related to LVDD were measured, and the results indicated significant changes in left atrial dimension (LAD), interventricular septal thickness (IVST), mitral septal, and lateral velocity among the three groups (P < 0.001) (**Table 3**).

**Table 3:** Characteristics of enrolled type 2 diabetics grouped by different levels of NLR.

NLR levels	Overall	Low	Medium	High	P
	n=855	n=214(0.601,1.316)	n=412(1.316,2.156)	n=229(2.156,15.622)	
Sex,n (Female %)	264 (30.88%)	82 (38.32%)	131 (31.80%)	51 (22.27%)	0.001
Age (year)	56.00 (47.00-62.00)	53.00 (43.00-60.00)	55.00 (46.00-60.00)	58.00 (52.00-63.00)	<0.001
Course of diabetes (year)	6.00 (1.00-13.00)	5.50 (1.00-11.00)	5.50 (1.00-13.00)	8.00 (3.00-14.00)	0.004
Smoking (%)	375 (43.86%)	88 (41.12%)	178 (43.20%)	109 (47.60%)	0.364
BMI (kg/m2)	24.96 (22.88-27.47)	24.97 (22.98-27.22)	25.17 (23.05-27.83)	24.68 (22.32-27.46)	0.093
SBP (mmHg)	131.00 (120.00-142.00)	132.50 (121.00-144.75)	131.00 (120.00-141.25)	131.00 (119.00-142.00)	0.701
DBP (mmHg)	81.00 (74.00-89.00)	81.00 (74.00-89.00)	81.00 (75.00-89.00)	80.00 (72.00-88.00)	0.173
HbA1c (%)	8.90 (7.30-10.70)	9.10 (7.10-11.22)	9.00 (7.50-10.70)	8.60 (7.18-10.30)	0.121
TBA (mmol/L)	3.40 (2.10-5.20)	3.00 (2.00-4.60)	3.40 (2.10-5.12)	3.60 (2.35-5.70)	0.096
SCr (umol/L)	67.40 (56.10-80.50)	64.50 (52.90-74.00)	66.80 (55.82-78.45)	74.00 (60.68-96.25)	<0.001
BUN (mmol/L)	5.68 (4.58-6.99)	5.26 (4.40-6.55)	5.65 (4.60-6.82)	6.09 (4.82-7.60)	<0.001
GFR (ml/min)	100.72 (89.09-109.72)	103.72 (96.34-112.73)	101.25 (91.08-110.85)	95.24 (69.81-105.17)	<0.001
UA (umol/L)	332.40 (272.70-403.15)	334.70 (266.95-414.15)	333.35 (272.70-398.03)	329.20 (277.00-403.75)	0.864
TC (mmol/L)	4.55 (3.82-5.30)	4.65 (4.03-5.48)	4.53 (3.82-5.33)	4.40 (3.58-5.17)	0.004
TG (mmol/L)	1.49 (1.03-2.32)	1.54 (0.99-2.69)	1.54 (1.05-2.30)	1.29 (0.97-2.11)	0.059
HDL-C (mmol/L)	1.02 (0.88-1.24)	0.98 (0.85-1.27)	1.04 (0.89-1.22)	1.03 (0.88-1.29)	0.584
LDL-C (mmol/L)	2.70 (2.10-3.36)	2.71 (2.21-3.33)	2.71 (2.17-3.41)	2.58 (1.93-3.27)	0.044
FBG (mmol/L)	7.50 (6.20-8.90)	7.30 (6.30-9.00)	7.60 (6.20-8.90)	7.30 (6.00-8.90)	0.626
AST(U/L)	20.00 (17.00-26.00)	21.00 (17.00-27.00)	20.00 (17.00-26.50)	20.00 (17.00-24.00)	0.051
ALT (median (IQR))	22.00 (16.00-34.00)	23.00 (16.00-37.00)	22.00 (16.00-34.50)	21.00 (16.00-29.00)	0.049
LAD (cm)	3.50 (3.30-3.80)	3.50 (3.20-3.80)	3.50 (3.30-3.80)	3.60 (3.30-3.90)	0.027
LVD (cm)	4.50 (4.20-4.80)	4.50 (4.20-4.70)	4.50 (4.20-4.80)	4.50 (4.30-4.80)	0.089
IVST (cm)	1.00 (0.90-1.00)	1.00 (0.90-1.00)	1.00 (0.90-1.00)	1.00 (0.90-1.10)	0.004
mitral septal e’ velocity (cm/s)	7.00 (5.80-8.20)	7.20 (6.00-8.90)	7.00 (5.90-8.20)	6.60 (5.35-7.75)	<0.001
mitral lateral e’ velocity (cm/s)	9.60 (8.00-11.60)	10.10 (8.20-12.00)	9.80 (8.10-11.80)	9.15 (7.50-10.83)	<0.001
LVDD prevalence (%)	47.8 (409/855)	35.51 (76/214)	49.27 (203/412)	56.77 (130/229)	<0.001

LAD, left atrial diameter; LVD, left ventricular diameter; IVST, interventricular septum thickness.

### 3.4 Correlation between NLR levels and the prevalence of LVDD

**Table 4** showed the crude and adjusted logistical models evaluating the correlation between different NLR levels and the prevalence of LVDD. Compared with the low-level NLR group, the OR value for the medium-level group was 1.764(1.255-2.478, P<0.001) and 2.384(1.626-3.497, P<0.001) for the high-level group. Additionally, after adjustment for the course of diabetes and HbA1c (defined as model 2), the OR value was 1.764 (1.255-2.478, P<0.001) and 2.384 (1.626-3.497, P<0.001) respectively, and the P-value for the trend was also significant (P<0.001). After further adjustment for age, sex, and serum creatinine (defined as model 3), the OR value for the high-level group, as well as the P-value for the trend, did not achieve statistical significance(P>0.05).

**Table 4:** Correlations between different NLR levels and prevalence of LVDD.

Exposure	Model 1	Model 2	Model 3
NLR group	OR (95%CI) P	OR (95%CI) P	OR (95%CI)P
Continuous NLR	1.326 (1.138-1.546) <0.001	1.278 (1.098-1.488)0.002	1.035 (0.947-1.13)0.449
Low	1	1	1
Medium	1.764 (1.255-2.478)0.001	1.704 (1.205-2.41)0.003	1.613 (1.095-2.375)0.015
High	2.384 (1.626-3.497) <0.001	2.170 (1.468-3.207) <0.001	1.332 (0.855-2.077)0.205
P for trend	<0.001	<0.001	0.219

Model1: non-adjusted; Modle2: HbA1c, course of diabetes were adjusted; Model3: Model2+sex, age, eGFR and BMI.

## 4 Discussion

Individuals with T2DM suffer metabolic irregularities, increased production of advanced glycation end products (AGEs), and inflammatory cytokines due to prolonged hyperglycemia. Together with comorbidities such as hypertension, obesity, and renal dysfunction, T2DM can result in varying degrees of damage to cardiac structure and function, hence promoting the development of diabetic cardiomyopathy and HF (5, 6, 15). LVDD, also called preclinical HF, serves as an early stage of the detrimental effects of diabetes mellitus on the heart and exhibits high prevalence among diabetic patients (16). In our study, the prevalence of LVDD among recruited hospitalized patients with T2DM was 47.8% (409/855), consistent with previous reports (17–21). Logistic regression analysis of patients’ clinical parameters revealed that the patient’s age, SBP, and SCr were independently associated with the prevalence of LVDD. In contrast to previous studies, this research did not find a positive correlation between FBG, HBA1c, and the prevalence of LVDD (20, 22, 23). A cross-sectional survey by Rishi T. Guria et al. found that the prevalence of LVDD among T2DM patients was 54%. The research indicated that the average HbA1c level was markedly elevated in the LVDD group relative to the non-LVDD group (11.07 ± 3.66% vs. 9.11 ± 2.95%, P = 0.004) (20). The results may be attributable to the baseline characteristics of our study participants, which predominantly comprised hospitalized T2DM patients with poor glycemic control. Data from outpatient or health screening populations could better elucidate the impact of differential glycemic control on LVDD prevalence. Moreover, the effects of hyperglycemia on the heart depend on elevated blood glucose levels and the duration of sustained hyperglycemia, which may play a more critical role. Consistent with findings from other researchers, our study suggests that diabetic patients with coexisting LVDD tend to have a longer disease course (24–26).

Inflammation plays a pivotal role in the onset and progression of heart failure. Research has shown that patients with LVDD exhibit significantly elevated levels of inflammatory markers, both systemically and in cardiac tissues, including TNFα, IL-6, and IL-1β, compared to individuals with normal cardiac function (27, 28). NLR, an inflammatory biomarker reflecting the body’s inflammatory state, has been associated with various cardiovascular diseases, such as coronary artery disease and heart failure (29). Additionally, increased NLR levels have been linked to diabetes and may serve as a marker of the low-grade chronic inflammation commonly observed in diabetes and its complications (30–33). Studies investigating the relationship between NLR and LVDD are scarce. In our study, the mean NLR was higher in patients with LVDD than those without LVDD. Furthermore, an increasing trend in LVDD prevalence was observed with higher NLR levels. However, after adjusting for confounding factors such as age, gender, eGFR, and BMI, this trend was no longer statistically significant (P for trend = 0.219). When stratifying patients into three NLR groups, significant differences in age and SCr were identified across the groups. This suggests that age and SCr may be confounding factors in the analysis. Nevertheless, this does not negate the potential utility of NLR as an inflammatory biomarker for identifying LVDD risk. Multiple factors, including glycemic control, renal function, and age, influence NLR, which should be considered when interpreting its clinical implications (7, 9, 34).

Our study highlights the critical concern of LVDD in individuals with T2DM, a group at elevated risk for heart failure. We present a reliable and generalizable dataset derived from data analysis from 855 patients. The study identified a significant finding: The prevalence of LVDD increased progressively with rising NLR levels, indicating the potential utility of NLR as a biomarker for identifying LVDD in patients with type 2 diabetes. Echocardiographic screening for LVDD is recommended for patients with elevated NLR levels, especially among the elderly with hypertension and declining renal function. For those diagnosed with LVDD via echocardiography, treatment strategies should prioritize antidiabetic medications with proven benefits in preventing or managing HF, particularly sodium-glucose cotransporter 2 inhibitors (SGLT2i) (35). Further research is needed to confirm whether SGLT2i have a therapeutic or preventive role in halting the progression of advanced HF in patients with diabetes and coexisting LVDD.

The study has some limitations that should be considered. First, a cross-sectional research design cannot establish a causal relationship between NLR values and LVDD. Additionally, the inclusion criteria could not allow the findings to be generalized to the broader population of individuals with type 2 diabetes. Including a more diverse range of individuals from community populations or outpatient settings in the study could improve the generalizability of the results. Lastly, the lack of data on patients’ use of antidiabetic medications and other diabetes complications prevents us from assessing their associations with NLR or LVDD prevalence.

## 5 Conclusions

Our research findings indicate a significant positive correlation between NLR values and the prevalence of LVDD in individuals with type 2 diabetes. NLR could potentially be used as a biomarker to allow patients’ risk stratification and detection of LVDD in early asymptomatic phases, significantly reducing the burden of heart failure. Further validation of the predictive value of the NLR on developing LVDD warrants robust prospective studies. Such efforts will not only enhance our understanding of the link between chronic inflammation and LVDD in diabetic individuals but also aid in the clinical management of diabetes-related cardiomyopathy.

## Data Availability

The original contributions presented in the study are included in the article/supplementary material. Further inquiries can be directed to the corresponding authors.
